# Prevention and Control of Human *Salmonella enterica* Infections: An Implication in Food Safety

**DOI:** 10.1155/2023/8899596

**Published:** 2023-09-11

**Authors:** Mwanaisha Mkangara

**Affiliations:** Department of Science and Laboratory Technology, Dar es Salaam Institute of Technology, P.O. Box 2958, Dar es Salaam, Tanzania

## Abstract

*Salmonella* is a foodborne zoonotic pathogen causing diarrhoeal disease to humans after consuming contaminated water, animal, and plant products. The bacterium is the third leading cause of human death among diarrhoeal diseases worldwide. Therefore, human salmonellosis is of public health concern demanding integrated interventions against the causative agent, *Salmonella enterica.* The prevention of salmonellosis in humans is intricate due to several factors, including an immune-stable individual infected with *S. enterica* continuing to shed live bacteria without showing any clinical signs. Similarly, the asymptomatic *Salmonella* animals are the source of salmonellosis in humans after consuming contaminated food products. Furthermore, the contaminated products of plant and animal origin are a menace in food industries due to *Salmonella* biofilms, which enhance colonization, persistence, and survival of bacteria on equipment. The contaminated food products resulting from bacteria on equipment offset the economic competition of food industries and partner institutions in international business. The most worldwide prevalent broad-range *Salmonella* serovars affecting humans are S*almonella* Typhimurium and *Salmonella* Enteritidis, and poultry products, among others, are the primary source of infection. The broader range of *Salmonella* serovars creates concern over multiple strategies for preventing and controlling *Salmonella* contamination in foods to enhance food safety for humans. Among the strategies for preventing and controlling *Salmonella* spread in animal and plant products include biosecurity measures, isolation and quarantine, epidemiological surveillance, farming systems, herbs and spices, and vaccination. Other measures are the application of phages, probiotics, prebiotics, and nanoparticles reduced and capped with antimicrobial agents. Therefore, *Salmonella*-free products, such as beef, pork, poultry meat, eggs, milk, and plant foods, such as vegetables and fruits, will prevent humans from *Salmonella* infection. This review explains *Salmonella* infection in humans caused by consuming contaminated foods and the interventions against *Salmonella* contamination in foods to enhance food safety and quality for humans.

## 1. Introduction

*Salmonella enterica,* an etiologic agent of salmonellosis in humans, is a Gram-negative, flagellated facultative anaerobes, rod-shaped bacterium of the *Enterobacteriaceae* family [1]. The *S. enterica* has more than 2600 serovars, taxonomically classified into six subspecies, sharing high sequence similarity [2, 3]. The six phylogenetic groups of *S. enterica* include *S. enterica* subspecies enterica (I), *S. enterica* subspecies salamae (II), *S. enterica* subspecies arizonae (IIIa), *S. enterica* subspecies diarizonae (IIIb), *S. enterica* subspecies houtenae (IV), and *S. enterica* subspecies indica (VI). Among the six subspecies, the *S. enterica* subspecies enterica is pathogenic, containing over 1580 serovars with adverse health effects on homeotherms [4, 5]. On the other hand, the nonenterica subspecies have economic importance to poikilotherms, and their pathogenicity is limited [6].

Salmonellosis, caused by subspecies of *S. enterica,* is a leading foodborne disease, with its health effects on many hosts, including animals, birds, fishes, and humans. Regarding clinical syndrome, *Salmonella* serovars belong to subspecies I (*S. enterica*) are divided into typhoidal *Salmonella* (*S.* Typhi and *S.* Paratyphi A, B, and C) and nontyphoidal *Salmonella* (NTS) [7]. Typhoidal *Salmonella* (*S.* Typhi and *S.* Paratyphi A) are found in humans and cause enteric fever. At the same time, *S.* Paratyphi B and C infect other animals (higher primates) with a syndrome similar to typhoid fever. Furthermore, the NTS typically causes gastroenteritis, and the frequency of causing invasive disease is dependent on host immunity. People with HIV infection, falciparum malaria, malnutrition, and other immunocompromised disorders have higher predisposing risk factors for invasive nontyphoidal *Salmonella* (iNTS).

The host-restricted or host-specific serovars mostly grow in one host. These include *Salmonella* Typhi, an etiological of typhoid fever in humans, *Salmonella* Abortusovis for sheep, *Salmonella* Gallinarum for chickens and other gallinaceous birds, *Salmonella* Choleraesuis for swine, and *Salmonella* Dublin for cattle. In industrialized countries, the NTS, including *S.* Typhimurium and *S*. Enteritidis, have the significance of self-limiting diarrhoea to a healthy individual and are associated with over half of reported *Salmonella* cases. The annual worldwide estimations of NTS account for 93.8 million enteric infections and 155,000 deaths [8]. The estimated total combined costs of medical care, loss of productivity, and premature deaths due to foodborne *Salmonella* infections of humans in the United States ranged from $4–11 billion per year [9]. Along with this, the NTS serovars have a global burden due to the broad vertebrate host range with public health consequences [8]. In the United States, the leading *Salmonella* serovars in humans, in descending order, are *S.* Enteritidis, *S.* Newport*, S.* Typhimurium*, S*. Javiana, and monophasic *S.* Typhimurium 4,[5],12:i- [10], while in the European Union are *S.* Enteritidis, *S.* Typhimurium, monophasic *S.* Typhimurium 1,4,[5],12, i-, *S.* Infantis, and *S.* Newport [5, 11].

The case fatality ratio (CFR) due to invasive nontyphoidal *Salmonella* (iNTS) investigated globally from 81 studies revealed 17 · 1% was for Africa, 14 · 0% for Asia, 9 · 9% for Europe, and 9 · 6% for the Americas [12]. A study by Stanaway et al. [13] reported that NTS is the most common bacterial bloodstream of higher incidence in sub-Saharan Africa (34.5 cases per 100,000 person-years). The mean all-age case fatality on the surveillance on the global burden of salmonellosis reported for sub-Saharan Africa in 2017 was 14.5%, with an estimated 13.5% for children < 5 years, 51.2% for those aged ≥ 70 years, and 41.8% for people with HIV [13]. *Salmonella* infection in humans induces focal diseases with life-threatening to immunocompromised individuals.

Invasive nontyphoidal *Salmonella* (iNTS) infection is not associated with diarrhoea; however, clinical features are similar to typhoid fever with symptoms of fever, respiratory difficulties, and hepatosplenomegaly [14]. Among others, the most prevalent iNTS circulating in Africa with multidrug resistance and high case fatality rates are *S.* Typhimurium sequence type 313 (*S.* Typhimurium ST 313) and *S.* Enteritidis sequence type 11 (*S.* Enteritidis ST11) [15]. The strains are multidrug-resistant (MDR) due to resistance to multiple antibiotics including ampicillin, chloramphenicol, kanamycin, sulphonamides, and trimethoprim–sulfamethoxazole plus resistance to fluoroquinolones and third-generation cephalosporins [15]. Like other pathogens, MDR *Salmonella* is insensitive or resistant to the administered antimicrobial medicines (structurally unrelated and have different molecular targets) despite being sensitive to them earlier.

The iNTS in Africa account for 4100 deaths annually, mostly among children <5 years of age [16]. A study observed 75.4% and 24.6% of *Salmonella* Typhi and nontyphoidal *Salmonella* cases identified out of 171 children screened for blood samples in rural districts in northern Tanzania [17]. In Malawi and South Africa, the cases of salmonellosis in children < 15 years of age are 54% and 32%, respectively, while in central Africa, the *Salmonella* spp. account for 73% of cases of bacteremia [18, 19]. The iNTS is estimated at 3.4 million patients annually, with an overall incidence of 49 cases per every 100,000 population globally [20]. The colossal burden is from Africa, where out of 535,000 cases of iNTS infection occurred in 2017 around the world 421,600 cases (79%) are from sub-Saharan Africa [21, 22].

*Salmonella* infection associated with consuming contaminated foods is a threat to human health. Beyond being sick, humans acquire MDR strains from contaminated foods of plant or animal origin facilitated by plasmid and transposon exchange and aid the circulation of MDR strains around the global population [23]. Despite the consequences mentioned above, *Salmonella* spread in foods hinders international trade and minimizes the sustainable assurance of food safety and security in some parts of the world. Conversely, it weakens partner institutions' economy in food processing and production [24].

In this review, the author discussed the causes of *Salmonella* infection, its pathogenic mechanism and how contaminated foods are vehicles for salmonellosis in humans. The fates of biofilms for *Salmonella* survival on different surfaces were discussed in the article. The interventions against *Salmonella* contamination in foods were also explained.

## 2. Literature Search

Searching for published articles describing causes and interventions for *Salmonella* infection in humans was done using electronic databases. The academic journal articles and ebooks were used to screen and synthesize information related to the topics of the article. The desk review literature was used to assess the scientific qualities of the selected studies from 1950 to 2023. The relevant issues were screened centrally for the topic under investigation. Professional sites, including the European Food Safety Authority (EFSA), the European Centre for Disease Prevention and Control (ECDC), Food and Drug Administration (FDA), and the Centers for Disease Control and Prevention (CDC), were used to gather information on *Salmonella*, an aetiology of the foodborne disease in human and animals. Nevertheless, electronic databases were used to synthesize information on preventing and controlling *Salmonella* contamination in foods to enhance food safety and quality for human consumption.

## 3. *Salmonella* Pathogenicity Mechanism

The ability of *Salmonella* to cause diseases depends on several factors, including the capacity for invasion (involving fimbriae, flagella, and effector proteins), bacterial load, genes related to virulence, and the evasion of host immune response [25]. Several genes related to virulence are located in *Salmonella* pathogenicity islands (SPIs), a large region of chromosomes that encode virulence-related genes. So far, 17 SPI have been described; however, the most studied are SPI-1 and SPI-2 [26]. The SPI-1 encoded type III secretion system (T3SS) is an invasion island in all *Salmonella* species and subspecies with genes for invading nonphagocytic cells. The SPI-1 forms a channel that allows a bacterium to inject effector proteins into the host cell cytosol during the intestinal phase of infection [27]. The injected proteins induce cytoskeleton rearrangement that allows the internalization of tight-bonded *Salmonella* on the epithelial membrane into the mucosa of the cells. During internalization, the bacterium is taken into the vacuolar compartment known as *Salmonella* containing vacuole (SCV). *Salmonella* blocks the fusion of the SCV with a terminal acidic lysosome, which constitutes an important intracellular defense strategy of a eukaryotic cell [28].

The SPI-2 encoded the T3SS which is expressed a few hours following invasion is related to the ability of the bacterium to survive in phagocytic cells and replicate within SCV in eukaryotic cells. Inside phagocytic cell (i.e., macrophages), SCV matures, ruptures, and disseminates *Salmonella* into the cytosol of reticuloendothelial cells (liver and spleen) through the circulatory system and induce a systemic phase of infection [29]. The other SPIs are mainly involved in macrophage survival, replication, production of proteins, adhesins, toxins, and fimbriae encoding [30, 31].

## 4. Vehicles of Salmonellosis

The primary route for *Salmonella* infection in humans is through faecal-oral transmission or ingesting contaminated foods, including beef, pork, chicken meat, eggs, milk, fruits, vegetables, and water [32]. Despite faecal-oral being the most predominant route of *Salmonella* infection, airborne transmission also occurs in individuals exposed to dust contaminated with *Salmonella* [33]. A study by [34] observed *Salmonella* infection through dust and aerosolized particles in some animals, including pigs. The *Salmonella enterica* serotype 4,[5],12:i detected in faeces and body fluid of weaned pigs was also found in the environment where the pig was raised [35]. The contaminated dust is taken through the pharynx, located posterior to oral cavity down into the stomach through the oesophagus. In a similar environment, *Salmonella* infection in chicks has been experimentally proven to occur by oral, intracloacal, intratracheal, intraocular, navel, and aerosol administration [36]. This is evidence of why staff working in animal houses without personal protective equipment (PPE) such as masks may be infected with *Salmonella*.

## 5. Prevalence of *Salmonella* in Foods

The prevalence of *Salmonella* in the developed and developing world can easily be traced back by assessing what serovars affect humans and are also isolated from contaminated foods. In 2018, in the European Union (EU), the estimated human infection cases due to *Salmonella* reached 91,859 people, equivalent to 33% of all foodborne outbreak illnesses [37]. Infected eggs were directly linked to 1581 *Salmonella* cases in Slovakia, Spain, and Poland [37]. The resistant *Salmonella* serotypes investigated from 807 retail meat samples in China from 2011 to 2016 observed 159 (19.7%) samples positive with *Salmonella.* Pork ranked the highest, followed by beef, while smoked pork was the least [38]. Among others, *S.* Enteritidis, *S.* Typhimurium, *S.* London, and *S.* Derby were the most prevalent. In Australia, between 2001 and 2016, Ford et al. [39] examined 990 *Salmonella*-reported cases. The results were 79% (778 cases) had been transmitted through contaminated food, while eggs and egg-containing foods were the most identified food vehicle of *S.* Typhimurium. The prevalence of *S. enterica* in foods and human cases in Mexico in 2017 reported 92013 cases of NTS, which were twice to seventh reported cases of *S.* Typhi (45,280) and *S.* Paratyphi A (12,458), respectively [40]. The *S.* Typhimurium was the most common serotype isolated from foods and human cases. A total of 459 different samples of foods were investigated in northern Taiwan between January 2017 and December 2019, revealing 117 food samples positive for *Salmonella*, and pork (64.1%) and chicken (29.1%) were the primary contaminated foods [41]. In the study, *S.* Derby (16.2%), *S.* Anatum (13.7%), and *S.* Agona (8.5%) were the prominent serovars. From 2001 to 2002 in Italy, serotyping showed that 50% of the isolates from raw poultry meat (9.9%), raw pork (4.9%), and processed meat (5.3%) belonged to the serotypes mostly isolated from humans [42]. The *S.* Typhimurium was a leading serovar, followed by *S.* Derby and *S.* Enteritidis. The *S.* Weltevreden reaches global importance due to seafood [5].

Sub-Saharan Africa accounts for 78.8% of all *S. enterica*, nontyphoidal cases globally, from an estimated 342,000 (5.9 cases per 100,000 people) occurred in 1991, increased to 535,000 cases (7.5 cases per 100,000) in 2017 [43]. Among other countries in the developing world, *Salmonella* spp. was detected in 25% of the tested buffalo meat in Egypt from November 2020 to June 2021, and *S.* Enteritidis (20.7%) was a leading serovar followed by *S.* Typhimurium (17%) [44]. Despite the serovars being of interest in buffalo meat, their resistance was observed against erythromycin, streptomycin, clindamycin, cefepime, and nalidixic by 100%, 98.1%, 88.7%, 77.4%, and 66%, respectively. A total of 154 NTS cases were reported out of 60 research from 13 North African and Middle Eastern countries, representing 24,023 tested food samples, revealing 1,324 NTS-positive samples [45]. *S.* Typhimurium (28.0%), *S.* Enteritidis (23.6%), and *S.* Kentucky (20.3%) were the most common serotypes in the tested food commodities. The prevalence of *Salmonella* in Ethiopia in 2014 in food animals of cattle, sheep, goats, and pigs was 7.07%, 8.41%, 9.01%, and 43.81%, respectively [46]. Like most regions, *S*. Typhimurium ranked higher in Ethiopia, followed by *S.* Mishmarhaemek, *S.* Infantis, and *S.* Hadar. In Nigeria, the prevalence of *Salmonella* in raw milk was higher (4.6%, *n* = 16) than in fermented milk (3.4%, *n* = 11) [47]. Similar findings were observed in raw fish (35%, *n* = 13) compared to fermented fish (9%, *n* = 1) contaminated with *Salmonella* spp. in Thailand [48]. The low pH is linked to the reduction of *Salmonella* in food and food sources following the above differences.  Table 1 presents *Salmonella* prevalence in foods from different countries.

The disparities between developed and developing countries make factors such as geographical location, environmental factors, the vigour of an infected host, and management system account for *S. enterica* in food sources. However, among others, *S.* Typhimurium and *S.* Enteritidis are still the prominent *Salmonella* serovars of public health worldwide, making countries expend many resources to overcome their infections.

## 6. The Fate of *Salmonella* Biofilm Formation

The biofilm formation is one major factor enhancing the colonization, persistence, and survival of *Salmonella* in a viable dormant state on biotic and abiotic surfaces [72]. The biofilm assembles surface-associated microbial cells enclosed in extracellular polymeric substances (EPSs) [73]. The EPSs are a slimy matrix comprising carbohydrates, proteins, and extracellular deoxyribonucleic acid (eDNA) [74]. The biofilm is among the adaptative mechanisms through which *Salmonella* survives environmental stress, including pH variability, osmotic changes, and host immune responses. Other adaptations are against disinfection, ultraviolet (UV) light radiation, antimicrobial agents, and metal toxicity [75].

Biofilm formation occurs in several stages: (i) attachment (reversible attachment during adhesion to the surface followed by irreversible attachment during the production of extracellular matrix and quorum sensing), (ii) formation of microcolonies, (iii) maturation with cellular differentiation, (iv) detachment, and (v) dispersion [76]. *Salmonella* produces fimbriae, curli, flagella, adhesion proteins, and capsules to attach to the surfaces (biotic or abiotic) during biofilm formation. Curli are involved in cell aggregation and surface adhesion, mediate host cell invasion, and are potent inducers of the host inflammatory response. The fimbriae allow the bacterial cells to colonize and attach to epithelial surfaces [77]. Biofilm formation has been implicated with *Salmonella* cell growth in close proximity, communication (quorum sensing, QS), and the production of autoinducers to regulate gene expression for survival, growth, resistance against antimicrobials, tolerance to desiccation, and pathogenesis. In microcolonies, bacterial cells grow, accumulate, and form mature biofilms significant for food contamination [78]. From mature biofilms, loose cells are sloughed off and converted into planktonic cells, which start the life cycle of a biofilm by attaching to new biotic or abiotic surfaces.

The functions of EPSs in assisting *Salmonella* to overcome environmental stresses are concentrating nutrients, inhibiting biocidal agents, and increasing hydration to surfaces. Biofilms are significant in *Salmonella's* spread and persistence in different fields, including medicine, the environment, and food industries [79, 80]. A study by [81] observed that 80% of *Salmonella* chronic infections are associated with biofilm formation, which induces recalcitrance to antibiotics and limits antibiotic efficacy against bacteria. In addition, biofilms enhance antibiotic resistance caused by the cross-combination of resistance genes of multiple bacterial species in contaminated foods [82]. The biofilms developed on food surfaces, food processing, and packaging equipment favour adhesion and multiplication of bacteria, including *Salmonella*, with an ultimate of threatening food quality and safety [83, 84]. The biofilm ruins food safety, enhances *Salmonella* colonization, and induces survival, persistence, and transmission to equipment that later contaminates foods during value-addition processes. In this regard, bacterial biofilms institute an important concern for the food industry and food safety authorities as a significant source of food contamination with pathogenic and spoilage microorganisms [84, 85, 86].

## 7. *Salmonella* in Animal Products

The most consumed animal products linked to foodborne illnesses affecting public health worldwide are chicken, pork, beef, eggs, milk, and seafood [87]. Raw foods can be contaminated through contaminated hands, water, manure, equipment in the abattoir, and drops from birds, reptiles, insects, and pets, to mention a few. For instance, contamination of animal meat occurring in the abattoir is associated with the skills of personnel in gut evisceration, carcass examination, handling, and the poor hygienic standards of the processing rooms [88]. In addition, the bacteria from contaminated animal products contaminate equipment in food processing facilities, ultimately providing unsafe products that can affect human health.

*Salmonella* contamination in animal products is also linked to how animals are reared and processed, from farm animals to market products. For example, the infected chickens are a constant source of infection through the vertical and horizontal transmission of *Salmonella*. Vertical transmission of *Salmonella* occurs after bacteria penetrate through the eggshell or by direct contamination of egg contents (albumin, vitelline membrane, and finally the yolk) before oviposition [89]. Subsequently, the chicks hatched from the contaminated eggs are the source of infection in the flocks. Such infected breeds have an imperative role in the prevalence and persistence of *Salmonella* in the flocks, with a threat to the food safety of eggs, chicken meat, and their products [90]. The horizontal transmission also occurs through housing facilities (e.g., other farm animals, old litter, contaminated cages, feeders, drinkers, farm workers-clothes and boots, and bedding material). A study by [68] observed *Salmonella* serovars in the environment of 15 laying hen farms, the same as those recovered from raw chicken meat and commercialized eggs. Thus, hygienic environments for rearing animals and *Salmonella*-free breeds in farms are critical for food safety.

Animal products, such as chicken, pork, cow meat, or seafood, may be contaminated by microflora along the processing stages from farm to slaughtering unit. Pigs can be infected during transport or the waiting period in lairage before slaughtering. Studies have suggested that lairage and the slaughterhouse environment are probably the major sources of *Salmonella* infections before slaughter [91]. During slaughter, not only carcasses of infected animals but also cross-contamination from the environment and other infected animals may occur. For instance, Berends et al. [92] reported that 29% of the *Salmonella*-positive carcasses of pigs were due to cross-contamination. The results agreed with other studies that observed cross-contamination by 30% of pig carcasses in the slaughterhouse [93].

Poultry carcass preparation involves different steps, including scalding, picking, evisceration, and chilling, to reduce the total microbial load on the carcasses [94]. Through these processing steps, cross-contamination is possible if a single carcass is contaminated with *Salmonella*. Several studies have indicated the chilling process as a main contamination point after examining the changes in bacterial diversity on carcasses after immersion chilling and in the chilling immersion water [94, 95, 96]. For example, during the chilling stage, the carcass temperature was reduced to 40°F (4.4°C) or below within 4–8 h of slaughtering to prevent microflora growth. However, the carcasses leaving the chiller had 37% of *Salmonella* incidences [90], while in the other processing stages, the incidence ranged from 10 to 20%. Regarding this observation, the chilling process aids pathogen attachment on the chicken skin associated with deep channels and crevices.

In storage facilities, contamination of eggs and eggshells has been identified as the major cause of foodborne *Salmonella*. The egg internally contaminated with *Salmonella* (during formation in the reproductive tract of an infected hen) is a threat to storage facilities, food handlers, and during food preparation. *Salmonella* from raw egg products such as mayonnaise, burgers, milkshakes, and ice cream has been associated with disease outbreaks worldwide. For example, in the United States, between 1985 and 2002, contamination of egg food products was identified as a source of 53% of all *Salmonella* cases reported to the Centre for Disease Control and Prevention (CDC) [97]. Therefore, raw foods purchased from stores and supermarkets should be prepared and handled well to avoid foodborne pathogens including *Salmonella*.

Based on One health perspective, *Salmonella* is a zoonotic pathogen affecting both humans and animals [98]. Bush meats and their associated products are reported to contain *Salmonella* thus the significant vehicle of salmonellosis in humans. The ready-to-eat (RTE) traditional dried and spiced meat made from bush meat, beef, chicken, or wild animals (biltong and jerky South African and United States dried meat, respectively) have been associated with salmonellosis cases in humans [99]. The prevalence of salmonellosis from 1973 to 1974 in biltong was 16% of all salmonellosis cases in native South Africans [100]. An epidemiological study on salmonellosis in London in 2008 identified 16 hospitalised people due to *S.* Typhimurium DT104, and out of them, 4 consumed biltong purchased from a South African food outlet [101]. The possibility of *Salmonella* contamination in biltong and jerky might be due to infected animals or contamination during slaughtering and processing. Marinating biltong (from lean strips of beef) with traditional spices (coriander, black pepper, salt, and vinegar) is dried at ambient temperature and humidity to lower water activity (*a_w_*) and inhibit the growth of microorganisms [102]. However, the use of nonthermal drying in biltong's production process raises concerns about the safety of the meat product.

Under normal conditions, bacteria are unable to multiply below a water activity (*a_w_*) of 0.85 [103]. Contrary to this, *Salmonella* is capable of remaining viable at *a*_*w*_ ≥ 0.94 [104]. Therefore, RTE dried meat products should all time be consumed under precautions against foodborne pathogens. Similarly, in beef jerky preparation, the heating step is involved to achieve the recommended 5-log reduction of foodborne pathogens [99]. However, at the infective dose ≤ 10^3^ (3-log), immunocompromised humans are at risk of salmonellosis. This makes biltong and jerky consumers around the world aware of the consequences that might occur when consuming such products that have not undergone proper heating and microbiological testing.

Seafood is accountable for a significant number of foodborne diseases and is of great concern to public health globally. Seafood contaminated with serotypes other than *S. enterica* subspecies enterica (subspecies I) has limited pathogenicity to humans. *Salmonella enterica* is excluded among the components of the normal flora of sea foods. However, the contamination of *Salmonella* in sea foods results from faecal contamination through polluted water, infected food handlers, or cross-contamination during production or transportation. Nontyphoidal *Salmonella* is among the frequent contaminant of seafood. For instance, in India, a study by [105] observed an incidence of 20.7% (*n* = 17, 82) of finfish group harbour *Salmonella*. Another study from Saudi Arabia reported that 28% (*n* = 14) of tilapia imported from India was contaminated with *Salmonella* while the whole eastern provinces of Saudi Arabia (including Thailand, Vietnam, Bahrain, India, and Myanmar) had incidence level of 39.9% (*n* = 89, 223) [106]. In the United States, *Salmonella* incidence level of 3.2% (*n* = 5, 156) has been reported in smoked and shellfish [107, 108], whereby in Iran, 2.9% (*n* = 2, 70) of fish samples, 4.3% (*n* = 3, 70) of shrimp samples, and 13.8% (*n* = 9, 65) of RTE fish samples tested positive for *Salmonella* [109].

Despite contamination of animal products along the value chain, *Salmonella* also spreads through the trade of live animals within and between countries. For instance, the spread of infection with *S.* Typhimurium in Europe resulted from the business of calves and parent and grandparent flocks in the poultry industries [110].

## 8. *Salmonella* in Fresh Produce

*Salmonella* proliferates and survives in plant tissue, including *Arabidopsis* (cabbage family) and tomatoes (*Solanum lycopersicum*), similar to animal tissues [111]. This observation was evident after detecting *Salmonella* in the thin lateral root of cabbage 3 h postinoculation of bacteria in irrigated water. Later, after 20 h, *Salmonella* was found in rhizodermal cells contaminating cells of the main root [112]. The study was the first experiment by Professor Herbert Hirt of the Perutz Laboratories in Vienna on plant tissue infection by the human pathogen. On the farm, plant products are exposed to *Salmonella* contamination through untreated manure, contaminated irrigation water, and wildlife contact and drops, particularly rodents, reptiles, and birds [113, 114].

*Salmonella* secretes periplasmic enzymes that break plant surface barriers efficiently [115]. Also, the level of ripening and wounding on plant surfaces makes *Salmonella* easily penetrate enzymes and facilitate entry and survival [116]. Therefore, to a greater extent, fresh produce such as tomatoes, beans, watermelons, papaya, lettuce, cucumber, alfalfa, and mangoes contaminated with irrigation water have been associated with *Salmonella*-related outbreaks [117]. According to the Centers for Disease Control and Prevention (CDC), it has been documented that in all pathogens related to food contamination investigated from 2006 to 2017, *Salmonella* alone contributes to 53.4% (55/103) of foodborne disease outbreaks, with 32.7% of it in fresh produces [118]. Thus, *Salmonella* in irrigation water is a critical factor for unsafe vegetables and fruits. The sources of contaminated irrigation water are partially treated wastewater and groundwater, mainly contaminated with the leaching of latrines and septic tanks or surface water.

Once released from the gastrointestinal tract (GIT) to surface water, either broad or restricted *Salmonella* serovar resists environmental stress. Therefore, environmental stresses such as a change in pH, temperature, nutrients, and ultraviolet radiation threaten *Salmonella* less. Under these circumstances, *Salmonella* can stay alive in water or soil for many days and contaminate the plant produce. *Salmonella* survives at pH and temperature ranges of 4.05-9.5 and 7-48°C, respectively; however, the growth temperature is 37°C. The broader temperature range for *Salmonella* survival and proliferation proves that the bacterium survives outside the animal host.

Interestingly, at about 25°C, *Salmonella* in a closed environment can survive up to 5 years in a phosphate-buffered solution. In plant tissue, *Salmonella* survives a hostile environment for up to 14 days after inoculation into plant tissue [119]. Among other reasons, the chances of surviving *Salmonella* on different surfaces are due to biofilm formation. A study by Gaertner et al. [120] detected *Salmonella* from water biofilm samples collected 23 days apart with the same repetitive sequence-based polymerase chain reaction (rep-PCR) profile. This observation is evidence of why contaminated water with *Salmonella* used for irrigation is a critical source of contamination to fresh produce. Harvested contaminated plants with *Salmonella* are the intermediate hosts that end up accessing a new niche on the GIT after being consumed by humans.

A study by Brandl et al. [121] observed the plant pathogen *Pectobacterium carotovorum* promotes the growth of *Salmonella* by macerating plant tissue and providing nutrients for *Salmonella* colonization. This study corroborates Zheng et al. [122], who found *Salmonella* internalized into tomato plant (*Solanum lycopersicum*) through stomata pores and wounded tissue; however, the colonization and survival ability differ with serovar involved and inoculum density. The power of *Salmonella* to escape the plant immune system in tomatoes and stay alive for about 14 days is similar to phytopathogens. The phytopathogens evade the host immune response by delivering effectors through type III, IV, and VI secretion systems (T3SS, T4SS, and T6SS), similar to *Salmonella* [123]. Underlying this observation, pathogenic bacteria infecting plants, animals, humans, and fish have various secretion systems representing major virulence determinants. Using secreted enzymes such as proteases, lipases, and pectate lyases, the bacteria degrade eukaryotic cell wall components and decompose host polymers. The secreted enzymes distributed to the environment are executed mostly by types I, II, and V secretion systems (T1SS, T2SS, and T5SS), while effector proteins are delivered by T3SS, T4SS, and T6SS into a host cell [124].

*Salmonella* transit from the field to a table occurs when products such as vegetables and sprouts are minimally processed during heating to maintain organoleptic properties. The RTE vegetables and fruits in supermarkets and grocery stores are minimally processed when washing alone is inadequate to preserve and sustain flavours, smell, taste, nutrients, and other desirable parameters; however, food safety to consumers is doubted.

## 9. *Salmonella* in Processed Food Products

Foods are altered during preparation by freezing, canning, baking, drying, heating, smocking, etc., to preserve organoleptic properties, inhibit pathogenic microbes, and prolong shelf life [37]. Despite these preparations, pathogenic bacteria such as *Salmonella* have been isolated from processed foods, including ice cream, cheese, mayonnaise, dry and fermented milk, chocolate, chicken nuggets, nut butter, frozen pot pie, and sandwiches [125]. Others are ready-to-eat foods (RTE), such as biltong, jerky, and salad vegetables that are precleaned and precooked ready for consumption [126]. In 2016, sushi, a Japanese food containing sesame was found positive for the *S. enterica* subspecies enterica serotype 11:z41:e,n,z15 by a private laboratory in the United Kingdom [127]. The findings were communicated to the public as a warning to the subsequent products. The trace-back and forward studies on the originality of the strain observed similar *S. enterica* serotype 11:z41:e,n,z15 reported in sesame exported from Greece to Germany had its origin in Sudan [127]. The evidence displayed how food commodities exported from one region are a vehicle of salmonellosis to another country.

A study on the diversity of serotypes in swine, poultry, and cattle products observed by Figueiredo et al. [128] revealed that out of 14 serotypes identified from 258 *Salmonella* isolates, the most prevalent were *S.* Typhimurium (32.6%, *n* = 84) followed by *S.* Enteritidis (10.1%, *n* = 26). The *S.* Kentucky on polythene bags used to wrap RTE foods in groceries and restaurants was found to resist environmental conditions at higher temperatures (25°C to 42°C) and higher pH values (7 and 8) due to biofilm formation [129]. Therefore, proper storage temperature (4°C and below) and pH (pH 4.2 and below) have a significant impact on the reduction of *Salmonella* in foods [130]. *S.* Enteritidis has been observed in dry egg yolk powder used to make mayonnaise, ice cream, noodles, and salad dressings [131]. In another study, it has been observed that low water activity (a_w_) inhibits the growth of microorganism [132]; however, *Salmonella* spp. have been found to increase heat tolerance at low water activity. The adaptations make *Salmonella* survive in low-moisture foods such as chocolate, milk powder, and peanut butter and frequently cause worldwide outbreak [133].

## 10. Water Activity (*a_w_*) and *Salmonella* Survival

Low water activity (*a_w_*) foods are foods that do not support the growth of microorganisms, including moulds, bacteria, and yeast [69, 134]. Unlike water activity, water that is not chemically linked to other substances is known as free water. Free water is an appropriate medium for the growth of pathogens. Free water is measured by the activity of water, which is the ratio between a solution's vapour pressure and pure water's vapour pressure at the same temperature. Pure water has *a_w_* of 1, and microorganisms cannot grow at this value. Adding nutrients gradually reduces the *a_w_* value and favours the growth of pathogens. Foods are considered safe at a low water activity of 0.83. However, *Salmonella* can survive up to *a*_*w*_ ≥ 0.94 on the shelf at room temperature [135, 136]. Despite the immune response against pathogens from human, still, *Salmonella's* infective dose to induce the disease range from 10^5^ to 10^8^ cells and ≤10^3^ in immunocompromised people [137]. Therefore, to maintain food safety for human consumption, all the ingredients and the environment used to process and make low-water activity foods, such as powdered infant formula, pasta, chocolate, peanut butter, spices, dried fruits, nuts, and snacks, should be *Salmonella*-free.

Initially, low-water activity (*a_w_*) foods were considered safe because of suppressed microbial growth due to their low *a_w_* value or because they undergo crystallization, dehydration, desiccation, and lipid oxidation, which are not favourable for microorganisms' survival [138]. However, this perception is wrong because salmonellosis outbreaks related to low-water activity foods are increasing yearly. For example, in 2001, the number of salmonellosis cases due to German chocolate accounted for 400 people. According to the European Centre for Disease Prevention and Control (ECDC) report of January 2022, the number of salmonellosis cases due to chocolates increased to 450 people [139]. A wide range of *Salmonella enterica* serotypes has been detected in low *a_w_* foods. A study by [140] identified *S.* Newport, *S.* Typhimurium, and *S.* Tennessee in wheat flour at the range of low *a_w_* of 0.45–0.46 with a survival time of 1 year at the temperature 20°C.  Table 2 presents *Salmonella* survival in selected low-water activity foods.

The extent of *Salmonella* survival in comparison to other Gram-negative bacteria depends on several factors, including temperature, *a_w_* level, food substrate, and serotype. The ability of *Salmonella* to survive in low *a_w_* also increases its heat resistance, and the presence of fat in the food matrix provides additional protective effects to the bacteria [150]. However, in the presence of sodium chloride (NaCl) concentrations between 3% and 4%, the development of *Salmonella* is usually inhibited [151]. The inhibitory action of salt increases with increasing storage temperatures.

## 11. Prevention of *Salmonella* Infections

Preventing *Salmonella* infections in humans requires more than one strategy. Among the strategies to be considered together to eliminate *Salmonella* contamination in foods and enhance food safety for humans are the following.

### 11.1. Biosecurity Measures

Biosecurity measures are the main factor in minimizing environmental exposure to *Salmonella* contamination and risks of *Salmonella* spread in animal houses [152]. However, prevention is difficult because of persistent faecal-oral conditions associated with healthy animals that shed out *Salmonella* bacteria without showing clinical signs. In addition, the *Salmonella* from infected animals can remain viable in the environment for six or more years.

*Salmonella* prevention in low-moisture and fermented foods of animal origins can be managed by having *Salmonella*-free animals. The animals include pigs, fowl, cattle, fish, goats, and sheep, to mention a few. The prevention of *Salmonella* infections in these animals requires multiple interventions because of the ability of the bacterium to survive environmental changes associated with more comprehensive ranges of temperature and pH. The strategies to employ *Salmonella* minimization in the animal house include cleaning and disinfection to prevent contamination of successive groups of animals. In slaughterhouses, the minimum time for animals to stay in pens is essential to reduce cross-infections and cross-contamination [152]. During the slaughtering of animals, a process of hide removal from animals should be correctly done to prevent an outside of the animal's skin from coming in contact with the fresh. While processing the meat, the processing unit and storage units should be separated, and disinfection should be routinely done and supplemented by inspecting the meat through microbiological tests.

*Salmonella* infection in live chickens is highly occurring in poultry houses through dirty feet, feathers, feedstuffs, and water. Therefore, to maintain the food safety of chicken products, including meat and eggs, there should be *Salmonella*-free breed and supply stocks of chickens in the house. Such chickens can be obtained by screening and vaccinating each batch of flocks. Free from *Salmonella*, flocks should be raised using hygienic feeds, wearing clean protective clothing for workers, and having rodents, reptiles, and bird-proof housing. Other means include disinfection of footwear and vehicles entering the poultry houses, clean water troughs, regular removal of droppings and litter, testing, culling, and disposal of sick or dead flocks. Decontamination using strong disinfectant is necessary to maintain a safer environment for each batch of flocks in the houses.

Prevention of *Salmonella* contamination in vegetables and fruits considers biosecurity measures similar to animals. The treatment of manure used on the farm to minimize bacteria associated with foodborne pathogens, including *Salmonella,* is essential [153]. Water for irrigation of vegetables and fruits must be free from pathogens since a bacterium such as *Salmonella* enters the plant through the stem system and stomata pore on the leaves. Workers should adhere to hygienic principles in food processing facilities before and after leaving the industry.

### 11.2. Isolation and Quarantine

The well-established isolation and quarantine are among the strategies for controlling *Salmonella* infection and subsequent persistent contamination in the farm environment [154]. During isolation, the asymptomatic individuals are removed from the general population. In cases where the health status of the incoming animals is not known or suspected to be inadequate, quarantine programs are necessary. Animals under quarantine should be frequently observed for illness or abnormal behaviour and should be screened for diseases before mixing with other animals on the farm [155]. Concurrently to this, introduced new animals for breeding, fattening, or any other purpose are first recommended to be kept in isolation facilities and screened for diseases before mixing with other animals on the main farm. The definite period for isolation of animals in the facilities varies according to the pathogens suspected. For instance, the cattle and pigs on arrival to the farm first undergo isolation for a range of 21 to 30 days, and their faecal samples have to be free from *Salmonella* at the end of the isolation period [156].

Quarantine animals live in isolation, 100 to 150 M from the rest of the animals. Farmers are advised to buy new animals from trusted sellers, screen for diseases, check for ectoparasites, and access the vaccination history of arrival animals into farms to know when to offer the next vaccine while keeping them under quarantine [154]. Occupation safety procedures for controlling the spread of diseases on farms necessitate farm staff attending quarantine animals to be different from other employees. Equipment used in the quarantine area should not be used, under any circumstances with the rest of the farm. Quarantine policies have managed Australia nation free from significant diseases of aquatic. The policies were designated to meet the international trade obligations of Australia that involve isolation in quarantine premises and prohibiting the importation of live aquatic species for commercialization [157]. In addition to this, the use of quarantine stations for livestock exported from Somalia to Middle Eastern countries reduced the spread of bacterial and viral diseases [158]. Animals undergo clinical examination, laboratory screening, and vaccination and then are left under quarantine for 21 days before issuing clinical certificates for exportation to Middle Eastearn countries. Therefore, freeing animals from pathogens including *Salmonella* is a competitive advantage for farmers to operate well in international trade.

### 11.3. HACCP Principles and Food Safety

Hazard analysis and critical control point (HACCP) are the logical system of food control based on prevention. HACCP emerged and evolved as superior to quality control when food companies voluntarily acquired knowledge and skills about food safety management. The HACCP ensures a safe food supply to consumers through standards that deal with food safety management. The standards are reviewed every five years to assess whether a revision is necessary to ensure that the standards remain relevant and useful to businesses. ISO 22000 derived from ISO 9000 is an international standard that specifies the food safety management systems in using HACCP principles to provide safe food products from any contaminant, including pathogens. Many countries have different agencies and parastatals that oversee the application of HACCP to avoid food safety disasters [159]. The governments impose the frameworks within which food safety issues can be managed. The frameworks include education and training on the management and causative of foodborne pathogens and standards of safe foods for humans. The training is routinely done for food industry practitioners, regulatory personnel, and supporting systems.

The success of HACCP has increased calls to regulators, politicians, and consumers to use effectively this management system to ensure food safety along the entire food chain from farm to table (Schlundt, 2002). Despite the use of HACCP in food industries still, foodborne outbreaks occur. However, the failure is not HACCP but rather the food industry's owners in cleaning and sanitation practices, lack of management awareness and commitment to providing resources and training to workers [160]. For instance, the Danish pork industry focused on abattoir interventions that largely reduced the numbers of *Salmonella* seropositive pigs delivered to slaughtering units by first using hot-water decontamination of carcasses, sanitary slaughter for farms with high *Salmonella* prevalence, and use of acidified feed for pigs meant for slaughtering [161].

Routine measuring of contamination in the food processing industry assesses the physical, chemical, and microbiological environments of the whole food chains. The strategy is important because the microorganisms are evolving, and their mutagenicity and antimicrobial resistance strains are a menace to the food industry and food security globally. Moreover, climate change continues to impact agricultural produce, and rampant water scarcity affects food production [162]. Therefore, food contaminants including foodborne pathogens are threat to food security and have negative impacts on human health. The HACCP identify critical control points (CCP) where control of food safety hazard must be applied such as destroying or eliminating vegetative pathogens to maintain the quality of the food products to consumers [163]. Thus, the HACCP strategies in keeping safe foods from farm to table are fundamental to all manufacturers, processors, retailers, and packers of varieties of plants, animals, and seafood products [164].

### 11.4. Animal Feeds

The presence of *Salmonella* spp. in many types of ingredients such as grains, oilseed meals, and fish meals has been reported in animal feed and the vehicle of *Salmonella* transmission to animals [165]. The most reported *Salmonella* serovars include *S.* Typhimurium, *S.* Montevideo, *S.* Hadar, and *S.* Tennessee [166]. Maintaining the food safety of animal products and eliminating *Salmonella* contamination in animal feed is significant for human health. Reduction of *Salmonella* contamination in animal feeds can be done through heat treatment, the use of organic acids, and other chemical preservatives [167]. It has been suggested that animal feeds under heat treatment of 80–85°C for a range of 2 to 12 min are sufficient to destroy *Salmonella* (and 0.8 water activity) [168]. However, in some circumstances, at 80°C depending on the strain is not sufficient; thus, other options such as additional chemical compounds such as organic acids are applied. Adding organic acids to animal feed changes its pH value (pH 4.5 and lower) and creates unfavourable conditions for the growth and survival of *Salmonella* [169]. Other short-chain fatty acids, such as acetic, propionic, and butyric acids, have all been shown to have an inhibitory effect on *Salmonella* growth.

Herbs and spices are plants with prebiotic activity that are used as feed additives of natural origin with beneficial effects on the health and performance of the animals [170]. Herbs and spices are added to animal feed as dried plants, extracts, or parts of plants (leaves, seeds, stem bark, root bark, etc.). The plants contain secondary metabolites with several biological effects including modulating the intestinal microflora and thus preventing the adhesion of *Salmonella* on the intestinal epithelial. For example, animal feed mixed with an extract containing active ingredients of cinnamaldehyde, capsicum oleoresin, and carvacrol enhances the growth of lactobacilli and so increases the ratio of lactobacilli to outcompete enteric pathogens [170]. Consequently, herbs and spices help to increase the resistance of the animals exposed to different stresses, and increase the absorption of essential nutrients, thus reducing the susceptibility of animals against pathogens.

Strong antibacterial efficacy of carvacrol and eugenol was observed against *Salmonella enterica* serotypes infecting turkeys [171]. Growth-promoting effects of feed supplemented with cinnamon, oregano, thyme, cayenne pepper, and citrus extracts were more efficient for broiler performance [172, 173]. Similar to this, pigs that received feed supplemented with garlic or rosemary essential oils had very minimal *Salmonella* cases with effective digestion compared to the control group that received plain feed with no supplements [174]. The fact is that herbs and essential oils mixed in animal feeds have antimicrobial activity with the characteristics of inducing lysis to microbial cell membranes. The lysed membrane increases permeability, leading to leakage of the cell contents and reducing the proton motive force, thus killing the microbes [174]. Under these aspects, herbs and spices are not just for appetite (cinnamon, cloves, cardamom, laurel, and mint) and digestion stimulants but also impact the physiological functions, ensure good health and welfare of the animals against diseases, and thus positively affect their performance.

### 11.5. Epidemiological Surveillance

Several countries have established national and regional surveillance systems on foodborne diseases to be aware, detect and respond rapidly to disease outbreaks and halt their spread. Countries employ serotyping as a universal language for laboratory isolate-based surveillance for *Salmonella* detection. However, the global consensus is to move towards whole genome sequencing (WGS) for routine surveillance and outbreak detection for *Salmonella* [175]. Integrating surveillance and collaboration across human health, food safety, and animal health specialists and the combined efforts of the food industries, regulators, and public health officials are essential for controlling *Salmonella* along the food chains [176].

Humans are mostly infected with *Salmonella* after consuming contaminated food products or water. Measures of preventing human salmonellosis necessitate achievements in hygienic environments of food sources (plants, animals, and seafood) and the whole food value addition chains. For instance, the global epidemiological studies and the national surveillance programme of salmonellosis to humans conducted from 1990 to 1995 among 191 WHO Member States identified *S.* Enteritidis, *S.* Typhimurium, and *S.* Typhi as the most frequently isolated serotypes from 104 countries responded to the studies. Poultry products were the vehicle of salmonellosis in European and American countries with Enteritidis being a frequently reported serotype followed by Typhimurium. In African countries, *S.* Typhi was the most reported serotype and common in countries with limited sanitary infrastructures [177].

Another observation from the global epidemiological study on *Salmonella enterica* serovars in animal-based products (beef, pork, poultry, and seafood) from five continents (Africa, America (North and Latin America), Asia, Europe, and Oceania) identified *S.* Typhimurium from all four assessed matrices and continents [5]. In the same study, poultry played a primary role in distributing *S.* Enteritidis to humans, while Anatum and Weltevreden serovars were frequently reported in beef and seafood, respectively. Such surveillance data on food contaminants are essential to medic and vet specialists in facilitating the identification of potential reservoirs for interventions. Regarding the reported serovars infecting humans, control programs and specific interventions are implemented to reduce the risk of salmonellosis in humans. Moreover, reported outbreaks provide critical information about how to control the spread of the disease and prevent similar events in the future. From epidemiological investigations, the source of infections that support specific sources of contamination and the need to monitor the effectiveness of the control measures are the critical components to all national public health and vet stakeholders to respond against salmonellosis in humans. Thus, among others, HACCP strategies to ensure safe food on the table for humans are significant to all food manufacturers, regulators, and farmers throughout the production continuum across the continents.

### 11.6. Farming Systems

Farming systems are categorized as intensive, semi-intensive, and extensive systems [178]. In intensive farming, the animals are fed in confinement with no access to graze. In semi-intensive systems, animals are kept in a house at night and fed, but allowed to scavenge and forage during the day in a fenced designated area. In an extensive (free range/pastoralism) farming system, the animals are let loose for grazing, rely on pasture feeding, and create opportunities to live natural life [179]. Despite animals kept in intensive systems having lower disease burdens with higher growth performance, feed efficiency, supplements, and reproductive performances yet the economic losses related to contaminated products from these animals persist. For instance, cumulative monetary loss due to nontyphoidal *Salmonella* (NTS) in Nigeria in 2020 was US $930,887,379 with approximately 50.9% (US $473,982,068) and 49.1% (US $456,905,311) from infected humans and animals (poultry sector), respectively [180]. The increased intensification of chickens in Arusha, Tanzania, was associated with 15% (6/40) of farms tested positive for *Salmonella* [181]. In addition, *Salmonella* prevalence in chickens raised in Africa under intensive farming systems for the deep litter to broilers and battery cages to layers ranged from 0.8% to 93.34% [182]. The observed disease incidences in intensive systems are mostly associated with unhygienic water, feeds or fomites (clothes, vehicles, and equipment) supplied to animals, and poor biosecurity measures. On the other hand, the exposure of these animals to any kind of stress including physical (fatigue or injury), physiological (heat, cold, thirst, and hunger), or behavioural (unfamiliar animal or environment) lowers their immunity and makes them very susceptible to pathogens including *Salmonella.*

Environments, where animals are raised, contribute to safe food products. Animals with high levels of environmental contamination are more likely to produce contaminated products and create greater public health consequences than animals with low environmental contamination [183]. For example, the incidence of *Salmonella* infection in dairy goats was 31.1% (*n* = 270), and the lowest infection was from goat herds under the extensive system (13.3%). The infection rates of 36.7% and 43.3% were from goats raised under semi-intensive and intensive production systems, respectively [184]. In extensive farming, animals access a wide area to scavenge/graze therefore less cross-contamination through feeds and water. Contrary to intensive farming systems, contaminations of feeds and water are high, which later affect the safety of food products along the value chain. The infected hens raised in cages are more exposed to contaminated environments and more likely to lay infected eggs and, subsequently, chicks which acquire *Salmonella* through the hen's reproductive system [185]. In an aquatic environment, contaminated seafood such as fish, shrimp, clams, mussels, oysters, crabs, lobsters, squid, cuttlefish, and octopus often are from contaminated water and surroundings where seafood is handled [186]. Thus, the role of the environment is the most significant criterion for achieving the short- and long-term benefits of safe food products from animals. Interventions in environmental hygiene for raising animals and proper biosecurity measures are the key strategies to reduce cross-contamination and infection of *Salmonella* in animals.

## 12. Control of *Salmonella* Infection

### 12.1. Use of Phages

Bacteriophages (phages) are viruses used to infect bacterial cells and use bacteria machinery to create new progeny [187]. Bacteriophages (phages) can be found in various environments where bacteria grow, such as soil, water, wastewater, and even faeces, indicating their ubiquity [188]. The lytic and lysogenic phages are named after their replication activity to a susceptible bacterium. The phage attaches to a susceptible bacterium, introduces its genome into the cytoplasm, and utilizes the bacterium ribosomes to manufacture its proteins during lytic activity. Upon lytic activity, the bacterium resources are rapidly converted to viral genome and capsid proteins, then assemble into multiple copies of original phages. Subsequently, the bacterium cell is lysed and dies passively or actively, releasing new phages to infect another host cell [189]. In the lysogenic replication activity, the phage attaches to a susceptible host bacterium, introduces its genome into the cytoplasm, and integrates into the bacterial cell chromosomes or maintains it as an episomal element. In both cases, the lysogenic phage genome replicates and passes to daughter bacterial cells without killing them [189].

The potential of phages as an alternative to antibiotics is due to their powerful bacteriolytic activity, host specificity, self-limiting properties, and ease of genetic manipulation [189]. Despite these, most of the phages are stable at a wider range of pH values, salt concentrations, and temperatures. Phages infecting typical enteric bacteria such as *Salmonella* spp. should be resistant to the acid environment of gastric juice which influences their stability, replication, and survival [190]. Similar to the observation, the broad-spectrum phages such as LPSE1 and LPST10 are mostly suggested against *S.* Enteritidis and *S.* Typhimurium in RTE foods because of stability and strong lytic ability at the pH range of 4-12 [191]. The lytic *Salmonella* phages against *S. enterica* serovars in a broiler are suitable biocontrol with a broad host range and effective in reducing established biofilms after 5 and 24 h of treatment following changes in expression patterns of the biofilm-associated genes (*adrA*, *csgD*, and *gcpA*) [192].

Using phages against bacteria in foods has revolutionized the increasing antimicrobial resistance against microbes. Studies have documented how effectively phages reduce bacterial counts in various foods, including meat, vegetables, eggs, processed foods, and animal skin [191]. The cocktails of phages are better in treatment against several bacterial strains than a single phage with its specific bacterium host. Therefore, it is also best to combine phages with a narrow host range with other phages and use them as phage cocktails for treatment. Despite the strategies above, the coevolution of phages and their bacterial hosts resulted in several inherent limitations for using natural phages in therapeutics [193]. The challenges underlying phage use against bacteria include restricted host range, moderate antibacterial efficacy, and frequent emergence of phage resistance. To solve the challenges, the advances in synthetic biology and genetic engineering provide phages with additional antibacterial efficacy while improving the safety profile and adaptability of the host range [193]. The engineered phages have a species-restricted host range and target only relevant pathogens while preserving the commensal microbiota. Nevertheless, these phages have receptor-binding proteins (RBP) that prevent bacteria from evading phage reactions by modifying cell wall-associated receptors. Another means of enhancing phage's antibacterial efficacy is by producing heterologous proteins that deliver biofilm depolymerase and capsule depolymerase, quenching enzymes, and cell wall hydrolase with lytic activity against bacteria [194].

Phage biocontrol in the food chain has become a fascinating natural and green technology used to attack pathogenic bacteria in various food products to enhance food safety and nutritional values in food industries [195]. The application of phages in food industries has been proven by the FDA and among others, the phage product ListexTM P100 and LPSTLL in combination with LPST94 were found to be effective against *Listeria monocytogenes* and *Salmonella* spp., respectively [196, 197]. The significant increase in *Salmonella* resistance against antibiotics and other antimicrobial products has made phages potential in inhibiting *Salmonella* colonization on food surfaces.  Table 3 demonstrates examples of phage types and their application in different foods.

Despite finding phages helpful in preventing pathogenic bacteria in several foods, there are challenges to food processors in using phages [212]. Previous studies have shown that after applying phages to food surfaces, the concentration does not substantially increase [212, 213]. From this observation, it has been suggested that the progeny phages cannot attack additional bacteria in foods. The challenge of increased bacteria can be minimized when the phage solution is more concentrated upon application on food surfaces to increase the chances of phages invading targeted bacteria. However, this may create an economic burden on the food processor in affording the required phage product from the manufacturer [214]. Nevertheless, the bacterial resistance against phages that have been repeated in controlling similar bacteria or a narrow range of bacteria was reported to be managed by engineered natural phages [192].

Endolysins are phage-encoded enzymes produced at the end of their lytic life cycle [215]. The ongoing trials on phage-encoded enzymes have shown the antibacterial activity of progeny phages against pathogenic bacteria after cleavage of the peptidoglycan layer of the bacterial cell wall. In food applications, the antibacterial activity of endolysins has been demonstrated against pathogenic bacteria contaminating vegetables, milk, and beef [216].

### 12.2. Vaccination

*Salmonella* Enteritidis and *Salmonella* Typhimurium account for 70% of all *Salmonella* infection cases in humans associated with eating contaminated food products of chicken origin, mainly meat and eggs [217]. Among several adapted serovars, *S.* Typhimurium remains the leading serovar in transmission to chickens, followed by *S.* Enteritidis. These broad serovars have significant health consequences for humans worldwide. Therefore, the intervention through mass vaccination of free from *Salmonella* chickens on farms remains a core measure in reducing the prevalence of *Salmonella* in live animals with its implication for food safety. The live attenuated vaccines in layer chickens effectively induce protection against bacterial diseases due to a strong humoral immune response compared to killed vaccines or whole bacteria extract [218]. However, using live vaccines in chickens requires more safety than other vaccines to prevent reinfection risks when the mutation occurs. Live attenuated *Salmonella* vaccines are preferred because they have a broader host immune response for protection against multiple serovars than killed or inactivated vaccines [219]. The effectiveness of live attenuated vaccines in the flock demonstrated the rise of antibody titers shortly after vaccination, a characteristic of its potent protection against pathogenic bacteria [220]. A study by Jia et al. [220] in the field trial observed that the first vaccination of AviPro *Salmonella* DUO in chickens revealed no shedding of vaccine strains on day 2 after immunization. In a similar study, vaccinated chickens had lower flock mortality and higher egg production performance than unvaccinated flocks throughout life and during the egg production period [213]. In another study, the *Salmonella* mutant strain live vaccine and the killed vaccine's booster dose displayed higher efficacy in protecting chickens against advanced fowl typhoid caused by *S.* Gallinarum [221].

Therefore, vaccinating animals in combination with other control measures is a way forward in mitigating salmonellosis in humans caused by eating contaminated food products of animal or plant origin [222].

The development of vaccines against invasive typhoidal and nontyphoidal salmonellosis in the human population has improved the lives of people around the globe. Live attenuated oral vaccine Ty21a (Vivotif) and the injectable Vi capsular polysaccharide (Vi CPS) vaccine (Typherix or Typhim Vi) have been designed to induce bacterial lysis and express cross-protection against both *S.* Typhi and *S.* Paratyphi. The Ty21a strain is genetically stable and can be prescribed only to children over 5 years of age because of the high dose of vaccine that is required to achieve immunogenicity. A three-dose injection of Ty21a with a Vi CPS boost induces broader protection against *Salmonella* and has been proven the most effective in Americans and European countries, while Vi rEPA is licensed in China against *S.* Typhi to young children [223]. The vaccinated animals tend to increase the productivity of milk and reduce live shedding and intestinal colonization of *Salmonella*. Vaccination stimulates an immune response, protects animals against *Salmonella* infection, and finally provides healthy animals and food safety benefits [224].  Table 4 refers to examples of live attenuated vaccines used to confer immunity against *Salmonella* infection in animals and humans.

### 12.3. Herbs and Spices in Food Products

The use of herbs and spices to prevent food spoilage from contamination by pathogens has recently increased in modern food industries [235]. Within this context, herbs and spices have been traditionally used by ancestors since immemorial [246]. In food processing industries, herbs and spices are used to fortify foods for therapeutical and nutritional purposes against different animal and human diseases worldwide [247]. Despite being preservatives in combating microorganisms in various foods, the spices and herbs are residual-free, enhance organoleptic properties, and maintain food safety from contaminants. Other advantages are improving fortified foods' quality and shelf life than purified chemicals [248]. For example, *Cinnamomum cassia* Presl is an additive in foods that contains essential oil with antioxidant and antibacterial properties enough to disrupt bacterial cell walls, including *S.* Typhimurium [249]. Other spices containing essential oils with similar properties are rosemary (*Rosmarinus officinalis*), clove (*Eugenia caryophyllata*), oregano (*Origanum vulgare*), savory (*Satureja montana*), common thyme (*Thymus vulgaris*), and red thyme (Thymus zygis) [250]. The plants are rich in essential oils and contain some main bioactive compounds, including flavonoids, phenolic acids, aldehydes, and terpenes [251]. *Punica granatum*, *Myrtus communis,* and *Thymus daenensis* were used in some African countries, including Tanzania, in folk medicine due to antibacterial, antioxidant, anti-inflammatory, and antiviral properties attributed to phenolics and flavonoids [252].

Herbs and spices are collected from different plant parts [253]. Herbs are harvested from the plant's leaves, while spices are the bark, seeds, fruits, berries, roots, flowers, aril, or pods [254]. The herbal yoghurt selectivity for Gram-negative and Gram-positive pathogenic bacteria is attributed to higher antimicrobial activity due to bioactive compounds, including peptides and organic acids [255]. Fortified foods with plant extracts may contain three main categories of bioactive compounds: phenolics, terpenes, and terpenoids or alkaloids with multiple actions against pathogenic microbes. Among others, the mechanisms employed by bioactive compounds of plants against pathogenic microbes in fortified foods include compromising the genetic machinery of bacteria and interference with cell movements by altering the cytoplasmic membrane. Others disrupt iron uptake pathways for bacterial functioning, disrupting cell membranes and proton motive force [256]. Plants such as *Salvia officinalis* and *Schinus mole* L. have antibacterial activity against *S.* Anatum and *S.* Enteritidis inoculated on minced beef meat. In addition, the plants cleared bacteria from refrigerated raw beef. In the other study, citrus essential oil incorporated in edible biopolymer film preserves fish fillets against *Salmonella* species [257]. Comparable to the above findings, essential oil from grape seed minimizes the population of *S.* Typhimurium in raw ground beef [258].

The shelf life of unpasteurized fruit juice is easily degraded due to microbial activity. However, the fruit juices from apple, pears, and melon juice fortified with an extract from lemongrass and geraniol are active against *Salmonella* spp., *Escherichia coli*, and *Listeria* spp. [259]. Cucumber salad and low-fat yoghurt added with mint oil demonstrated higher activity against *S.* Enteritidis. Comparable to other essential oils, oregano oil is effective against *S.* Typhimurium on tomatoes and eggplant [260]. Most herbs and spices contain phenolics with lipophilic characteristics important for disrupting membrane permeability and osmotic balance of bacterial cells, which eventually lead to leakage of nucleic acids, amino acids, ATP, and ions. Essential oil from oregano successfully inhibited *S.* Enteritidis in vegetable salad and mayonnaise, where egg yolk powder is a main ingredient [261].

### 12.4. Probiotics and Prebiotics

The consumption of probiotics and prebiotics may prevent *Salmonella* colonization in the gut. Probiotics are live, nonpathogenic microorganisms mainly isolated from fermented dairy products and the faecal microbiome [262]. The probiotics include lactobacilli, bifidobacterial, and other lactic acid-producing bacteria (LAB). The availability of adequate probiotics in the gut of a human infected with *Salmonella* is among the control strategies for the adhesion of *Salmonella* on epithelial cells of the GIT [263]. Probiotics are bacteria that confer a health benefit to a host because of their successful competition with pathogens, stimulation of host immune responses, and increased gastrointestinal pH after anaerobic fermentation of carbohydrates [264]. Among cultured probiotics from either source, the *Lactobacillus acidophilus* inhibits the development of invasive pathogens such as *Salmonella* spp. after secreting lactic acid, which lowers the intestinal pH [265].

Prebiotics are fermented food ingredients that promote the growth or activity of a limited number of bacteria in the host colon for their health benefits [266]. Sometimes prebiotics are considered foods for bacteria species, including genera of *Lactobacillus* and *Bifidobacterium*, which are beneficial for the health and well-being of a host. Therefore, the utility of prebiotics to bacteria species is essential for providing nutraceutical and nutritional value to a host. The significant aspects of prebiotics include the selectivity of microbiota associated with health-promoting effects, resistance to digestion, and fermentation by intestinal microbiota [267]. Moreover, prebiotics promote beneficial bacteria in inducing biofilm attachment on the epithelial cells of the gut, which then aids in inhibiting pathogenic bacteria from adhesion to the gut [268]. From this observation, consuming prebiotic-rich foods has been proven to be the best intervention against pathogenic bacteria in the gut of a host [269].

### 12.5. Nanoparticles

The use of nanoparticles (NPs) as a carriers in targeting the site of pathogen entry or functioning on different body surfaces of an organism has been critical in recent years. The study observed that 90% of gastrointestinal infections occur at the mucosal surfaces of the epithelial layer [270]. The NP breaks the gastrointestinal barriers that hinder the drug's efficiency across the mucosa surface. The obstacles to drug delivery inside the host include the acid pH of the stomach, enzymes at varying concentrations, chemical compounds (i.e., glutathione), temperature, and exogenous and endogenous stimuli [271]. The NPs as the carriers to the sites of infection are directly related to a particle size critical in the drug delivery system.

Nanoparticles are solid, colloidal particles ranging between 1 and 500 nm in diameter; however, in a nanomedical application, the size is less than 200 nm. Depending on the classification of NPs, there are metal NPs, lipid-based NPs, carbon-based NPs, and polymeric NPs [272]. The polymeric NPs are colloidal with a size ranging between 50 and 500 nm and are efficient for oral application. In addition, the polymeric NPs efficiently cross the intestinal mucosa barrier of the GIT and facilitate the uptake of antigen-processing cells [273].

The plant parts such as the leaf, bark, root, and stem extracts are effective reducing and capping agents during the green synthesis of silver NPs (AgNP) [274]. In fighting against diseases, NPs are used as a carrier of plant extracts, antibiotics, or vaccines and enhance mobilization to the target area of infection inside the host. The study by [275] observed that AgNP mediated by *Ferula ovina* Boiss extract was more effective against Gram-negative *S.* Typhimurium and *E. coli* in vitro using the disk diffusion method. The more bactericidal effect of AgNPs is enhanced by their small size, which provides a larger surface area for interacting with the bacterial cell membrane. The AgNP attached to the bacterial membrane delivers its content (i.e., plant extract, antibiotic, or vaccine) and affects microbial functioning, including permeability and respiration.

Polymeric NPs such as chitosan nanoparticles (CNP) have been studied as a vaccine carriers for oral delivery of antigens against *Salmonella* [225]. The oral-delivered, synthesized *Salmonella* CNP is made from crude outer membrane protein and flagellin extracts of *S.* Enteritidis were evaluated against *S.* Enteritidi*s* and *S.* Heidelberg loads in broiler birds. A study observed that on day 3 postchallenge with *S.* Enteritidis and *S.* Heidelberg, the birds vaccinated with 500 *μ*g and 2000 *μ*g CNP had higher serum IgG than the control group. On average, the CNP vaccine at doses above 500 *μ*g induces an anti-*Salmonella* antigen-specific immune response in the broiler [276]. Additionally, a study by Acevedo-Villanueva et al. [226] evaluated the protective effect of the *Salmonella* CNP vaccine in broilers using in-ovo vaccination, revealing the vaccine's stability in acidic conditions.

The polymeric NP, such as CNP, breaks the barrier that prevents penetration and functioning of the drug to the target site by (1) resisting stomach acidic and alkaline pH, (2) slowing releasing the antigen for continued stimulation of the immune response, (3) being easily degradable, (4) being easier to be uptaken by antigen-presenting cells to ensure antigen mobilization and presentation, (5) being stable at room temperature, and (6) acting as an adjuvant on their own [225].

The information from previous studies and the ongoing research on CNP revealed the system suitable for oral delivery of *Salmonella* vaccine antigens to control *Salmonella* infection in poultry. Furthermore, the NP coupled with antibacterial agents in equipment, feed, and water are among the suitable preventive measures against *Salmonella* spread in foods to enhance human safety.

The combination of NPs with antibiotics delivered orally to humans against *S.* Typhi has revolutionized treatments for typhoid fever and offered numerous advantages, including no risk of developing resistance to the pathogen, lower cytotoxic effects, and no adverse health risk to a host [277]. Concurrently to the observation, the advanced targeted nanocarriers of carbohydrates polymers (e.g., chitosan), lipids (e.g., liposomes and niosomes), and metals (e.g., AgNP) have proven to be effective in treating typhoid fever via the oral route when coupled with antibiotics with poor diffusion across the intestinal mucosal [277]. The nanocarriers disrupt bacterial cellular organelles, DNA, enzymes, mitochondrial matrix, and lysosomes, resulting in increased bacterial permeability and finally bacterial cell death [278]. Additionally, NPs improve antibacterial therapy efficacy by enhancing antibiotic localization to the pathogenic cell and modulation of drug-pathogen interaction to overcome antibiotic resistance [279].

## 13. Antibiotic Growth Promoters and Multidrug-Resistant *S. enterica*

Many countries around the globe have burned the use of antibiotics as growth promoters. Contrary to developed countries, some drugs previously used as growth promoters in animal husbandry are still used to manage diseases in developing countries. For instance, fermented waste from tetracycline production (isolated from *Streptomyces* bacteria) was used in chickens as a source of vitamin B12 [280]. In Tanzania, tetracycline is still used against bacterial diseases; however, there is evidence of a higher amount of tetracycline residue, especially in poultry products [281]. An increase in tetracycline residue is associated with higher doses of drugs administered to chickens against diseases by unskilled farmers. A study by Jahantigh et al. [282] from Iran observed that the most prevalent type of drug resistance was tetracycline at 95%, while gentamycin was the least, with 21.7% in inhibiting *Salmonella* spp. This is evidence that bacteria have built resistance against tetracycline, and the misuse of the drug, primarily by unskilled farmers, continues to expand the range of bacterial resistance to other drugs. Similar to the above findings, avilamycin, avoparcin, flavomycin, monensin, and salinomycin are also used by some African countries to increase chick growth rates [283]. The underperformance of health specialists in providing awareness to people on the proper use of antibiotics against human and animal diseases creates a burden on medical and veterinary care with great setbacks to the drug discovery industry. The multidrug-resistant bacteria predispose more danger to the lives of animals and humans when the first drugs exhibited ineffective against some microbes. The everyday emerging multidrug-resistant strains around the global populations of humans and animals require research and academic institutions to look for more innovative strategies to overcome the disaster. Microbes interchange resistance genes located on bacterial plasmids and transposons for adaptation against particular classes of antibiotics or unrelated drugs upon the misuse of drugs. Therefore, a combination of strategies against *Salmonella enterica* serovars should be employed to reduce dependency on antibiotics. Prebiotics, probiotics, bacteriocins, phytoncides, organic acids, phytogenics (herbs and spices), bacteriophages, and immunostimulants are among the control measures against *Salmonella* [284].

Several bacteria, including *Salmonella,* that were susceptible to several antibiotics tend to resist drug reactions after the mutation, thus conferring resistance. The trace-back and forward investigations showed that some bacteria infecting humans are resistant to antibiotics and have similar trends of resistance to animals. A study by White et al. [285] investigated the human infection with ASSuT- (ampicillin, streptomycin, sulfonamides, and tetracycline) resistant *Salmonella* I 4,[5],12:i:- had its source from beef, chicken, and turkey. The least effective antibiotics against *Salmonella* isolates from buffalo in Egypt were erythromycin (100%, *n* = 53), streptomycin (98.1%, *n* = 52), and clindamycin (94.3%, *n* = 50) [46]. However, the low-rate resistance against amikacin, imipenem, gentamicin, cefotaxime, meropenem, ciprofloxacin, and enrofloxacin could be related to low-frequency use in veterinary fields [44]. In line with this study, Xu et al. [286] observed *S.* Rissen isolated from swine products resistant against tetracycline, streptomycin, trimethoprim-sulfamethoxazole, chloramphenicol, sulfisoxazole, and ampicillin. As reported by Su et al. [287], the higher resistance to the drugs above is attributed to the extensive use of tetracycline to feed animals and the cross combination and alteration of genes occurring in the bacterial genome with resistant traits.

A study in South Africa on what makes chickens kept under intensive care resistant against antibiotics revealed 32.1% (52/162) of poultry farms contain virulence genes (*misL*, *orfL*, *pipD*, *stn*, *spiC*, *hilA*, and *sopB*) with resistance trait against antibiotics used to treat salmonellosis in humans [288]. Typhoidal salmonellosis patients in Tanzania who were also HIV/AIDS cases were found to be resistant to ampicillin, co-trimoxazole, and chloramphenicol and sensitive to ciprofloxacin [17]. However, the resistance pattern to ciprofloxacin increased from 0.0% in 2009 to 15.4% in 2012 in the Democratic Republic of the Congo (DRC) [289].

Due to the resistance posed by *Salmonella* in the first-line drugs such as fluoroquinolones (FQs), the fluoroquinolone-resistant *Salmonella* species have been stated by the WHO as a bacterium of critical priority in research and development of new antimicrobial agents since 2017 [290]. Additionally, the drugs recommended to treat *Salmonella* infections in the United States, such as FQs (ciprofloxacin), extended-spectrum *β*-lactams (cephalosporin), and macrolides (azithromycin), have shown patterns of resistance against *S.* Indiana [15]. Therefore, establishing valuable national surveillance systems through comprehensive population genomic studies to trace the originality and evolution of antimicrobial-resistant serovars affecting both animals and humans is critical for medic and vet specialists.

## 14. Conclusions

Contaminated food products with *Salmonella* predispose consumers to risks of foodborne disease. The higher significant risks of salmonellosis are observed in street-vended traditional fermented foods worldwide. Regarding this, street vendors involved in processing and selling foods are a public health concern. They are frequently exposed to *Salmonella*, and studies have observed that the bacterium survives on human hands for more than three hours after contamination [126]. Humans, frequently infected with *Salmonella*, can develop colon cancer. The *Salmonella AvrA* protein stimulates the Wnt and STAT3 signalling pathways that induce the development of colonic tumour cells [291]. In supporting this, Mughini-Gras et al. [292] observed that *Salmonella* manipulates host cell signalling pathways and facilitates colon cancer development in genetically predisposed mice.

Nevertheless, infants, elderly, and immunocompromised persons infected with *Salmonella* develop severe complications with higher death rates. This burden has spiralled over costs from managing bacteria, the vehicle of disease, to treating salmonellosis in humans. For instance, an estimated $4-11 billion is used for medication, loss of productivity, and deaths of humans in the United States each year. In addition, studies have observed that the most prevalent *Salmonella* serovars in humans include *S.* Enteritidis and *S.* Typhimurium, which are also common in poultry. Therefore, preventing *Salmonella* contamination in animals, vegetables, and fruits is a foremost strategy for reducing salmonellosis and promoting the safety and quality of foods for human consumption.

Prevention and control of *Salmonella enterica* infections in humans are still challenging due to the broader number of *Salmonella* serovars that affect homeotherms. The prevalence of *Salmonella* infections differs significantly between geographical areas. The reasons include climate variability, consumer habits, food harvesting and processing technology, and land-use farming practices [293]. For instance, the serovars, such as *S.* Enteritidis and *S.* Typhimurium, are distributed worldwide, while others have specialized areas. In addition, *S.* Weltevreden is confined to Asia, while *S.* Anatum, *S.* Newport, and *S.* Eastbourne are most prevalent in Ethiopia [294]. Similar to these challenges, treating nontyphoidal *Salmonella* infection differs from typhoidal *Salmonella*. The frequent use of antibiotics to treat nontyphoidal *Salmonella* infection increases the relapse of infection and prolongs the gastrointestinal carrier state duration. The nontyphoidal salmonellosis is a self-limiting disease, and antibiotics do not affect the clinical signs of diarrhoea and fever, contrary to iNTS. Despite the abovementioned limitations, developing multidrug-resistant (MDR) *Salmonella* isolates complicates the treatment and management of several serovars infecting homeotherms. More or less, no single vaccine is effective against all the different forms of *Salmonella*.

The pathogenicity of *Salmonella* is known; however, the evolution in different serovars to survive the drug reactions creates concern in studying resistant genes attributed to drug resistance from each serovar. For example, fluoroquinolones have been recommended to treat *Salmonella* spp. in poultry, humans, and animals [295]. However, the growing fluoroquinolone resistance to *S.* Typhi strains has increased the utilization of cephalosporins and azithromycin against salmonellosis in South Asia. Furthermore, *S.* Enteritidis and *S.* Kentucky, S. Choleraesuis, S. Senftenberg, and S. Oranienburg have recently been reported resistant to ciprofloxacin, nalidixic acid, and azithromycin [296]. Therefore, the trends in multidrug-resistance serovars of *Salmonella* emerging in first-line drugs necessitate professional bodies, academic and research institutions, and food processing industries to find better interventions for dealing with such biological bombs. The above strategies discussed in this review can help mitigate *Salmonella* contamination in foods to promote human food safety. However, the need for local and international policies and laws to strictly control the trade of live animals, plants, and animal products within and between countries is suggested. Countries abide to trade policies and regulations differently, and sometimes corruption becomes a setback in preventing and controlling pathogens circulating in food products, including *Salmonella*. Therefore, epidemiological studies should be conducted from time to time to trace the most prevalent *Salmonella* serovars within and between countries, find the vehicles for the pathogen distribution and suggest control measures. The communicated epidemiologic surveillance report will aid in resisting and burning trades of contaminated food products or live animals from respective countries.

## Figures and Tables

**Table 1:** *Salmonella* prevalence in food sources from selected regions of the world.

No.	Sampled year	Food commodity	% prevalence	Positive samples	Country	Reference
1	2022	Pork (dry sausage)	54.5%	6/11	Italy	[49]
2	2016-2018	Pork	22.6%,	33/146	Romania	[50]
3	2013-2014	Chicken	19.8%	111/560	Iran	[51]
4	2015-2016	Vegetable samples	21.5%	87/405	Malaysia	[52]
5	2016-2019	Bovine, goat meat	10.5% 5.98%	19/181 7/117	China	[53]
6	March–November 2019	Pork	15.1%	219/1441	China	[54]
7	2016	Chicken	51%	102/200	South Africa	[55]
8	2002-2005	Food samples	0.009%	105/11516	Morocco	[56]
9	2015	Table eggs	7.7%	3/39	South Africa	[57]
10	2017-2018	Sandwiches	17.9%	36/201	Burkina Faso	[58]
11	2021-2022	Chocolate products	17.2% 16.6% 43.1% 6.6%	26/151 25/151 65/151 10/151	Belgium France United Kingdom Germany	[59]
			9.9%	15/151	Ireland	
12	2007-2008	Raw red meat and meat products	23.6%	/144	Algeria	[60]
	2015	Chicken skin Mixed chicken meat Frozen chicken breast fillets	64% 60% 52%	14/22 37/62 13/25	Egypt	[61]
13	2010-2012	Turkey	52.9%	9/17	Morocco	[62]
		Chicken	20%	17/86		
	2005-2006	Chicken breast, legs, liver, gizzard	10%	58/576		[63]
14	2014 2013	Seafood Portions of minced meat	23.9% 10.7%	11/46 6/56	Tunisia	[64, 65]
15	2015-2019	Eggs	33%		European countries	[66]
16	2001-2005	Almonds	0.87%	81/9274	California, USA	[67]
17	2015-2016	Chicken	26.67	36/135	Colombia	[68]
18	2016-2019	Reported outbreak	14.9%		Brazil	[69]
19	2015-2016	Chicken meat	18.1%	49/270	Singapore	[70]
20	2014-2015	Beef	7.5%	18/240	Malaysia	[71]

**Table 2:** *Salmonella* survival in low-water activity (*a_w_*) foods.

*Salmonella* serotypes	Foods	*a_w_*	Survival time	Reference
*S.* Enteritidis	Halva	0.18	>8 months under refrigeration	[141]
*S.* Agona, S. Enteritidis,	Peanut butter products	0.2–0.3	24 weeks at 5-21°C	[142]
*S.* Anatum, *S.* Enteritidis PT 9c, *S.* Enteritidis PT 30,	Almond kernels	0.4	12 months at 19.4 and 24°C	[143]
*S.* Typhimurium	Peanut butter	0.65–0.69	1 year	[144]
*S.* Anatum, *S.* S. Senftenberg 775W, *S.* Newport, *S.* Typhimurium, and *S.* Tennessee	Wheat flour	0.46–0.45	1 year at 20°C	[140]
*S.* Typhimurium DT104	Egg powder	0.2–0.3	8 weeks at 13 or 37°C	[145]
*S.* Eastbourne	Milk chocolate	0.41	>9 months at 20°C	[146]
*S.* Typhimurium	Peanut butter fondant	0.65–0.69	1 year	[147]
*S.* Anatum, *S.* Enteritidis PT 9c, *S.* Enteritidis PT 30	Raw nut	0.4	12 months at 20.4 and 23°C	[148]
*S.* Napoli, *S.* Enteritidis, *S.* Oranienburg	Cocoa butter	0.2	21 days at 5 or 21°C	[149]

**Table 3:** Examples of phages applied against *Salmonella* spp. into various foods.

Phage	Foods	Reference
SalmonFREE^R^	Broiler chicken via drinking water	[198]
UAB_Phi78, UAB_Phi87, UAB_Phi20	Chicken breasts	[199]
wksl3	Chicken skin	[200]
PhageGuard S^TM^ (previous Salmonelex^TM^)	Boneless chicken things and legs	[201]
P7	Raw and cooked beef	[202]
FOI-E2	Hot dogs, sliced turkey breast, and chocolate milk	[203]
Five phages	Chicken skin	[204]
BCP-1	Fermented soya bean	[205]
P22	Whole and skimmed milk	[206]
SalmoLyse^R^	Chicken, tuna, turkey, cantaloupe, and lettuce	[207]
SalmoFresh^TM^	Chicken breast fillets, broccoli, cantaloupe, and strawberries	[208, 209]
SJ2	Raw and pasteurized cheeses	[210]
Salmonella typing phage	Chicken skin	[211]

**Table 4:** Examples of live-attenuated *Salmonella* vaccines.

Name of the vaccine	Administration route of the vaccine	Reference
Poulvac^R^ ST	Sprayed to 1-day-old chick, followed by a booster after the age of 2 weeks through drinking water	[225]
Vaxsafe^R^ ST	Sprayed to 1-day-old chick, followed by a booster through drinking water after an age of 2 weeks	[226, 227]
AviPro^R^ Megan^R^ Vac1	Sprayed to one-day-old chick, followed in drinking water after 2 weeks, again in drinking water after 16 weeks of age	[228]
SALMOVAC® SE	Sprayed to 1-day-old chick, followed by drinking water after 7 days of age, then repeated after 7 weeks of age	[229]
Vaxsafe^TM^ STM1 *aroA*	Introduced into the eyes of a 1-day-chick, followed by drinking water after 6 weeks, then repeated after 10 weeks of age	[220]
AviPro *Salmonella* DUO	Administered to 1-day-old specific pathogen-free (SPF) chicken	[220, 230]
Gallivac^R^ SE	Orally delivered in chickens	[231, 232]
Nobilis® SG 9R	Delivered subcutaneously to a 6-week-old chick, then after 14 weeks of age	[233, 234]
TAD *Salmonella* vac® E	Delivered to 1-day-old chick, followed after 6 weeks of age, then after 16 weeks of age	[222, 235]
rOmpF and OMVs	Intramuscular against *S.* Enteritidis in humans	[236]
DIVA vaccine	Intranasal against *S.* Choleraesuis and *S*. Typhimurium	[176, 237]
Ty21a (Vivotif)	Oral administration against *S.* Typhi and *S.* Paratyphi B in humans	[238]
Vi CPS (Typherix or Typhim Vi)	Injectable administered against *S.* Typhi in humans	[238]
Vi CPS	Against *S.* Typhi in humans	[223]
Vi rEPA	Against *S.* Typhi in humans	[239]
Vi CRM197	Against *S.* Typhi in humans	[223]
Formulation of Vi rEPA and Vi TT	The preclinical trial against *S.* Typhi in humans	[238]
CVD909 *S.* Typhi	Against *S.* Typhi in humans	[240]
*S.* Paratyphi A CVD 1902	Intranasal route against *S.* Paratyphi A in humans	[241]
*Salmonella* Newport SRP	Injection route against *S.* Newport in cattle	[242]
SC54 vaccine	Intranasal route against *S*. Choleraesuis in swine	[243]
Enterisol® Ileitis	Via drinking water against Salmonella spp. in swine	[244]
Salmoporc®, IDT Biologika	Through ileocecal lymph nodes against *S.* Typhimurium	[245]
